# Age-specific incidence of need for long-term care for men and women in Germany 2015: Cross-sectional study comprising 82 million people

**DOI:** 10.12688/f1000research.129434.1

**Published:** 2023-01-27

**Authors:** Luisa Haß, Sabrina Tulka, Thaddäus Tönnies, Annika Hoyer, Rebecca Palm, Stephanie Knippschild, Ralph Brinks

**Affiliations:** 1 Witten/Herdecke University, Faculty of Health/School of Medicine, Chair for Medical Biometry and Epidemiology, Witten, 58448, Germany; 2 Leibniz Center for Diabetes Research at Heinrich Heine University, Institute for Biometrics and Epidemiology, German Diabetes Center (DDZ), Düsseldorf, 40225, Germany; 3 Bielefeld University, Medical School OWL, Biostatistics and Medical Biometry, Bielefeld, 33615, Germany; 4 Witten/Herdecke University, Faculty of Health/School of Medicine, School of Nursing Science, Witten, 58448, Germany

**Keywords:** epidemiology, ageing, long-term care

## Abstract

**Background: **With the growing number of older people, the number of people in need of long-term care is increasing, too. Official statistics only report on the age-specific prevalence of long-term care. Therefore, there is no data on the age- and sex-specific incidence of the need for care at the population level for Germany available.

**Methods: **Analytical relationships between age-specific prevalence, incidence rate, remission rate, all-cause mortality, and mortality rate ratio are used to estimate the age-specific incidence of long-term care among men and women in 2015. The data is based on the official prevalence data from the nursing care statistics for the years 2011 to 2019 and official mortality rates from the Federal Statistical Office. For Germany, there is no data on the mortality rate ratio of people with and without a need for care, which is why we use two extreme scenarios that were obtained in a systematic literature search to estimate the incidence.

**Results: **The age-specific incidence is about 1 per 1000 person-years (PY) in men and women at the age of 50 and increases exponentially up to the age of 90. Up to about the age of 60, men have a higher incidence rate than women. Thereafter, women have a higher incidence. At the age of 90, women and men have an incidence rate of 145 to 200 and 94 to 153 per 1000 PY, respectively, depending on the scenario.

**Conclusion: **We estimated the age-specific incidence of the need for long-term care for women and men in Germany for the first time. We observed a strong increase, leading to a huge number of people in need of long-term care in higher age groups. It is to be expected that this will result in an increased economic burden and a further increased need for nursing and medical staff.

## Introduction

With the growing number of older people and the age-related increasing impairment of independence and activities of daily living through illness or cognitive and communicative abilities, the number of people in need of care also increases. ^1^^–^^4^ To quantify the extent of long-term care, commonly the prevalence, which represents the proportion of individuals in a population in need of care and the actual care situation, is used. ^5^^,^^6^ In addition, the prevalence is frequently used to project future numbers of frail people in need of long-term care to form the basis for economic strategies and political decisions. Due to the fact that prevalence data is a composed estimate, which strongly depends on the rate of new cases of need for care and mortality, projections could be much more precise and comprehensive if exact information on incidence and mortality were used. In addition to being used as a suitable measure within projections, incidence can be used to assess the risk of needing care (e.g. age-spanning comparisons of the risk of requiring long-term care or comparisons between sexes). For questions of resource allocation, the age-specific incidence can be used to estimate at which age the risk of requiring care is particularly high and which sex in an age group is at higher risk. It can thus serve as a basis for calculations within different models for projections of future care needs.

Nevertheless, data on the incidence of the need for long-term care or the risk of developing need of long-term care are scarce for Germany. The Socio-Economic Panel (SOEP) found that for people aged 65 and over, the risk of needing long-term care increases by around 0.5 percentage points with each additional year of life. ^7^ This finding indicates a linear increase in risk with age. In the SOEP, data from a representative annual repeated survey of private households between 1984 and 2018 were evaluated. However, only people who are cared for on an outpatient basis and not within nursing homes were considered. Estimates of risk or the incidence of the need for long-term care at the population level are not yet available for Germany and would have to be collected through complex, lengthy and cost-intensive longitudinal studies. Therefore, another method of estimating incidence is required, as its calculation appears to be considered as an opportunity that leads to a more precise and comprehensive estimation of future prevalence.

One way of estimating the incidence is to use the illness-death model. This method determines the age-specific prevalence, taking mortality into account. As such, the model quantifies the relationship between the incidence rate, mortality rate, remission rate, and prevalence. With this relationship, it is possible to estimate the age-specific incidence rate at the population level. The aim of this article is therefore to estimate the age-specific incidence of long-term care using the illness-death model as the basis of further projections of future numbers of people in need of care. As a result, incidence rates for men and women were presented, allowing them to be used as a first prudent trend.

## Methods

The data used for this study were retrieved from the official statistics about the need for long-term care in Germany published by the Federal Statistical Office. These are issued every two years and provide information on persons with long-term care insurance. ^5^^,^^8^^–^^11^ The data was recorded as part of the national law in Germany (SGB XI) and includes information on people in inpatient, semi-stationary or outpatient care who received financial support ^5^ in the German population. This “Pflegestatistik 2019 - Pflege im Rahmen der Pflegeversicherung Deutschlandergebnisse” uses the following definition of the need for long-term care as a basis: “People who experience impairment of their independence due to physical, cognitive or psychological impairments or health-related stresses or requirements, which they cannot compensate for independently and are therefore permanently – probably for at least six months – dependent on help” and are assigned to care grades one to five (§ 14 Abs. 1 SGB XI). ^5^

For the period from 2011 to 2019, the age- and sex-specific number of people in the total population and the number of people in need of care were documented in two-year steps. The corresponding prevalence in different age groups (<15 years, 15-59 years, and in 5-year age steps from 60 years onwards up to 90+ years) were reported. To get prevalence data for every age, we logit-transformed the prevalence provided by the Federal Statistical Office and modeled it via multiple linear regression with calendar time ( *t*) and age ( *a*) as independent variables separately for men and women.

Within this analysis we used the illness-death model and the related partial differential equation (1), which specifies the change in prevalence in the course of calendar time and age. Thereby the analytical relationship between age-specific prevalence ( *p*), incidence rate ( *i*), remission rate ( *r*), mortality rate ratio ( *R*) (i.e., mortality rate of persons with need of long-term care divided by the mortality rate of persons without need for long-term care) and all-cause mortality rate ( *m*) ^12^ was described:

$$\left(\frac{\partial }{\partial t}+\frac{\partial }{\partial a}\right)p=\left(1-p\right)\left\{i-m\frac{p\left(R-1\right)}{p\left(R-1\right)+1}\right\}-\mathrm{rp}.$$ (1)

By rearranging the partial differential equation, the estimation of the incidence of long-term care can be calculated using the age- and sex-specific prevalence and all-cause mortality as well as the expected mortality rate ratio and remission as assumptions:

$$i=\frac{\left(\frac{\partial }{\partial t}+\frac{\partial }{\partial a}\right)p+\mathrm{rp}\;}{1-p}+m\frac{p\left(R-1\right)}{p\left(R-1\right)+1}.$$ (2)

The calculations were carried out for men and women separately. The corresponding age-specific mortality rates ( *m*) were determined as a function of the calendar year based on the mortality rates of the Federal Statistical Office. ^13^^–^^15^ Due to incomplete data availability, assumptions have to be made for the mortality rate ratio (R), which is not yet known for Germany at the population level. Hence, the information for *R* was derived from existing literature on long-term care which documented mortality rates in certain subgroups. ^22^ To account for the lacking information on mortality rates in long-term care, two different mortality rate ratios were used (as a constant factor) within the calculations to enable the assessment of a range of potential incidence rates. The lowest and highest values reported were selected and used in two scenarios for the incidence rate estimation. In scenarios 1 and 2, mortality rate ratios *R* = 3.2 ^16^ and *R* = 1.17 ^17^ have been chosen, respectively. The two different scenarios of R offer the possibility to determine a lower and an upper limit of the age-specific incidence rate for women and men.

In addition, assumptions about the remission rate were necessary for the analysis. Since dependency on long-term care in old age is often seen in connection with multimorbidity, which results in particular from chronic somatic or mental illnesses, ^4^ the need of long-term care was defined as a chronic and irreversible condition in this analysis. The remission rate *r* was therefore assumed to be zero. However, this assumption is supported by the recently published insurance report. In this report, a permanent termination of the need for long-term care (not due to death) of 0.3% in 2021 is stated. ^18^

Presentations of the results are given exclusively and by way of example for the year 2015 (which is the middle of the reporting period of the prevalence data) as age-specific incidence rate per 1000 person-years.

All calculations were performed in the freely accessible, open-source statistical software R Version 4.1.0 (The R Foundation for Statistical Computing). The underlying data and the source code for analysis of prevalences are available in the permanent, freely accessible online repository Zenodo. ^19^

## Results

**Figure 1.**
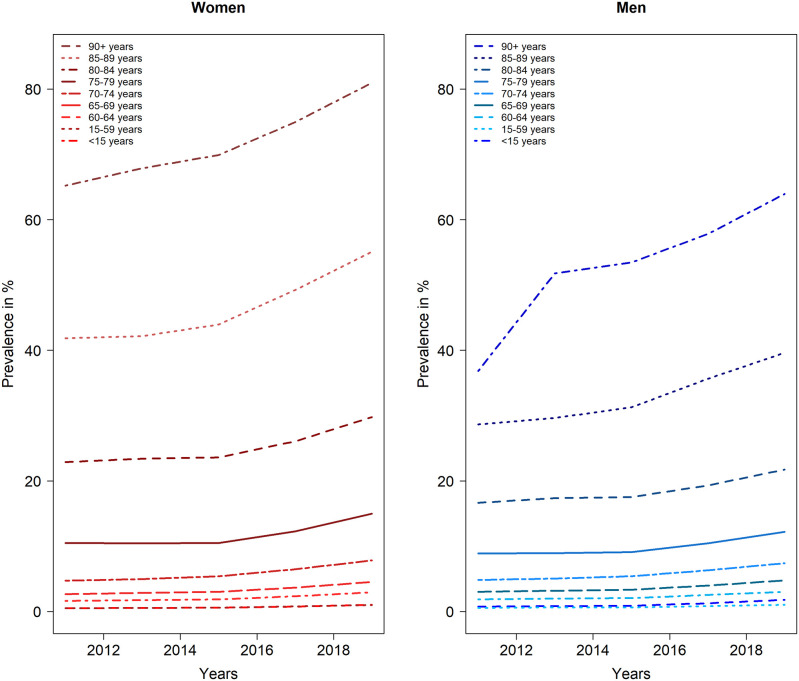
Line chart for the prevalence (in %) of the need for long-term care from 2011 to 2019; stratified by sex. Representation of the age groups from <15, 15-59, from 60 in 5-year intervals up to 90+ (Data basis: care statistics from the Federal Statistical Office for the years 2011-2019).

The underlying dataset showed a steady increase in the number of people in need of long-term care in Germany from 2.5 million in 2011 to 4.1 million people in 2019. Corresponding prevalences in the total population are 3.1% and 5% in 2011 and 2019, respectively. ^5^^,^^8^ Figure 1 shows that the prevalence of long-term care was higher among women aged 70-74 than among men. In addition, the prevalence strongly depends on age. In particular, from the age group 75-79 years, there was a larger “jump” in the need for care (to over 10 percent) compared to the younger age groups.

**Figure 2.**
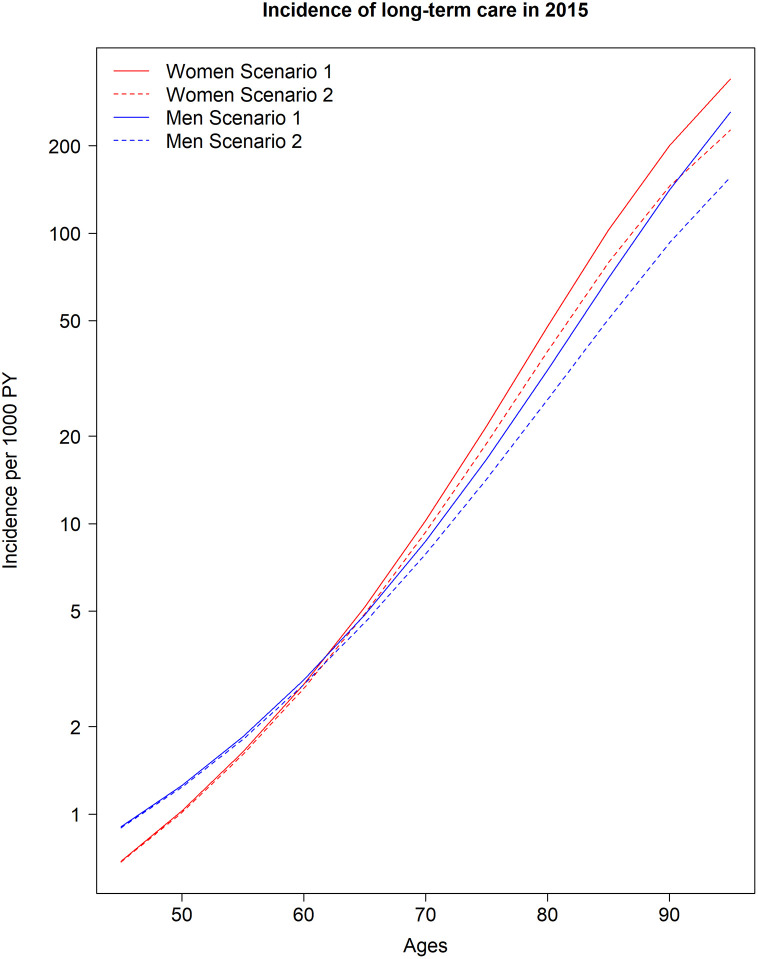
Logarithmic age-specific incidence rates per 1000 person-years in two scenarios depending on age, sex and mortality rate ratios ( *R*) for the year 2015. Scenario 1: *R* = 3.2; Scenario 2: *R* = 1.17.

For 2015, we found an exponential increase in the incidence rate in the age range of 50 to 90 years for both sexes (see Table 1 and Figure 2). Up to the age of about 65 years, men show a higher incidence rate for long-term care than women of the same age. From the age of about 65 years, the incidence of needing long-term care is higher for women than for men. With regard to the two scenarios of the mortality rate ratios, the incidence rate for a 50-year-old woman in scenario 1 ( *R* = 3.2) was 1.03 per 1000 PY and 1.01 per 1000 PY in scenario 2 ( *R* = 1.17). For men of the same age, there was a more than 20% higher incidence rate of 1.30 per 1000 PY in scenario 1 and 1.24 per 1000 PY for scenario 2. While in the lower age groups the differences between the two scenarios are small, the estimates between the scenarios differ with increasing age for both sexes. Men at age 90 had an incidence rate of 153 and 94.4 per 1000 PY in scenarios 1 and 2, respectively. For 90 year old women the corresponding values were 200 and 145 per 1000 PY in scenarios 1 and 2 ( Table 1, Figure 2).

**Table 1.** Incidence rate per 1000 PY in two scenarios for 2015 by sex, age and mortality rate ratios ( *R*) separately. Scenario 1: *R* = 3.2, scenario 2: *R* = 1.17 for all ages.

Age (Years)	Scenario 1 ( *R *= 3.2)		Scenario 2 ( *R *= 1.17)	
	Men	Women	Men	Women
**50**	1.30	1.03	1.24	1.01
**60**	3.08	2.80	2.81	2.71
**70**	9.62	10.3	7.95	9.38
**80**	38.3	47.7	27.2	39.2
**90**	153	200	94.4	145

## Discussion

The present analysis shows the first estimation of the age-specific incidence of the need for long-term care in Germany at the population level, stratified by sex. This incidence rate can be used to support economic and political planning in long-term care. Proper application of a partial differential equation related to the illness-death model provided an estimate of the incidence rate, taking into account a complete survey of the prevalence of the need for long-term care in Germany comprising the data of 82 million people. In contrast to the observation of a linear increase in risk, ^7^ our estimated rates show an exponential increase with increasing age for the year 2015 in both scenarios 1 ( *R* = 3.2) and 2 ( *R* = 1.17) for both sexes. Women from the age of about 65 years had a higher incidence of needing care than men of the same age.

Due to legislative amendments in definition of long-term care, changes in data collection and a deficient data situation, this analysis is subject to some limitations that must be taken into account when interpreting the results:

The care reform implemented in 2017 led to an adjustment of the definition of the need for care in replacing the existing care levels (I-III) by care grades (1 to 5). Although this change results in a more comprehensive and complete record of people in need of care, it also led to a larger number of people in need of care for this analysis. Some of the increases shown in Figure 1 might be due to this redefinition. In addition, changes in the context of data collection by the Federal Statistical Office must be taken into account. In particular, the number of people in need of care increased as a result of the first-time registration of people without a level of care and with significantly limited everyday skills in 2015. Moreover, the current care report 2019 integrated data from outpatient care services (according to § 71 paragraph 1a SGB XI) into the survey, which was previously not taken into account. Over time, these continuous adjustments may lead to a system-related increase in the prevalence of the need for long-term care, which means that the interpretation of the calculated risk might not be correctly represented. Due to these structural changes and the lack of information on the total number of persons with compulsory insurance in the reports, it was not possible to calculate the prevalence by different levels of care.

In addition to the data-based limitations listed above, further uncertainties must be taken into account due to the lack of information on the incidence calculation. In particular, the missing (real) mortality rate ratio, which is required for the calculation of the age- and sex-specific incidence of the need for long-term care, had to be replaced by considering extreme value scenarios from a literature search, which means that the incidence rates can only be narrowed down to a value range (lower and upper limit). Due to the lack of data on the subject of “mortality in need of long-term care” in Germany, a more precise estimation of the incidence remains impossible. This gap could be closed by transparent and comprehensive presentations of results in studies on aging and the need for long-term care, which should also include mortality. For a comprehensive result presentation of this analysis, incidences over time (general and stratified by sex) are available on Zenodo (Figure 3 ^20^ and Figure 4 ^21^). Due to lacking data on the mortality rate over time, these should be seen as possible trends only.

For further use of the results presented, consideration should be given to the fact that the remission rate in long-term care was set to zero. In general, and depending on existing comorbidities, a return of people in need of care to independence can possibly occur. However, a remission happens rather seldom than the termination of care due to death. ^18^ This care report ^18^ is the only published source of the remission rate in long-term care. It should be taken into account that this data is limited to one insurance, thus population-level data could not be used for this analysis. In accordance with the definition for determining the degree of need for care, according to § 15 SGB XI, the associated temporal classification of the need for care and based on the information from the available care report ^18^ we suppose that remission may occur rarely, but it is not impossible. Especially, the extent of the underestimation of remission in the first level of care cannot be quantified at this point in time due to the insufficient data on the improvement of independence in this low level of care. Additionally this subgroup was excluded in the care report. ^18^ An incorrect assessment of the need for help over a period of six months and the associated remission rate could not be taken into account as well. Therefore the remission rate *r* was assumed to be zero in our work. This leads to the fact that the calculated incidence rate can perform as the lower “limit” for the risk of needing care. As equation (2) shows a non-zero remission rate *r* > 0 would lead to an additional increase in the incidence rate. If remission is possible in grades with a low need for care, among patients in need of care who have diseases with an increased likelihood of remission, or in younger patients, the estimates of the incidence rates shown in this analysis represent a lower limit. However, given the lack of data, a quantitative statement about the influence of remission on the incidence estimation is not possible. In order to close the gaps in future estimates, data collection involving the relevant specialist sciences is still necessary.

## Conclusion

By carrying out this analysis, we were able to use an indirect method to estimate the age-specific incidence for men and women in Germany. Our findings indicate an exponential increase in the incidence rate with age in both women and men, which should be accounted for in future estimations on resource planning in long-term care.

We have performed a calculation of incidence rates, offering the possibility to predict future prevalence based on more extensive and precise information than a simple extrapolation of prevalence by itself. With existing data gaps regarding the epidemiology of long-term care in Germany, only a calculation of trends in incidence rates was possible. This prediction, based on an increasing number of new cases of long-term care and increasing overall mortality (due to the expectation of an increasing number of people over 65 years of age around the year 2030), might result in an expected prevalence that exceeds previous predictions.

## Ethics approval and consent to participate

The underlying data are aggregated and no individual subject can be identified. The data stem from a public data source (Federal Statistical Office). According to German National Laws, in this case no ethics approval and no consent to participate are necessary.

## Consent for publication

The underlying data are aggregated and no individual subject can be identified. The data stem from a public data source (Federal Statistical Office). According to German National Laws, no consent for publication is required.

## Data Availability

Publicly available datasets were analyzed in this study. The underlying prevalence data can be accessed from the “Pflegestatistik - Pflege im Rahmen der Pflegeversicherung Deutschlandergebnisse“ from the years 2011 to 2019 (see literature link included in the text or https://www.statistischebibliothek.de/mir/receive/DESerie_mods_00000940 ). Age- and sex-specific prevalence data were extracted from Tab. 1.2 for women and calculated for men. Mortality data were obtained from the official homepage of the Federal Statistical Office: https://service.destatis.de/bevoelkerungspyramide/ with the assumption of a moderate development of birth rate, life expectancy and migration (G2L2W2). Definition of sexes within this analysis was used in accordance to the definition in the published data. ***Figures:*** The figures presented within this analysis based on the above mentioned data, were created by the authors of this article. Zenodo: Estimation of incidence of need of long-term care from its prevalence in Germany, https://doi.org/10.5281/zenodo.6856273. ^19^ Zenodo: Age dependent incidence rates (1), https://doi.org/10.5281/zenodo.7440397. ^20^ Zenodo: Age dependent incidence rates (2), https://doi.org/10.5281/zenodo.7440523. ^21^ Zenodo: Age dependent incidence rates - literature search, https://doi.org/10.5281/zenodo.7440598. ^22^ Data are available under the terms of the Creative Commons Attribution 4.0 International license (CC-BY 4.0).
