# Supplementary material for: Age-specific incidence of need for long-term care for men and women in Germany 2015: Cross-sectional study comprising 82 million people
Source: F1000Res. 2023 Jan 27;12:102. [Version 1] doi: 10.12688/f1000research.129434.1 (PMC10043629; doi:10.12688/f1000research.129434.1)
Supplement: Peer review history

# Peer review: Reviewer response for version 1

**DOI:** 10.5256/f1000research.142119.r167164

**Published:** 2023-03-27

**Authors:** Emiel Hoogendijk

**Affiliations:** 1 Department of Epidemiology & Data Science, Amsterdam Public Health research Institute, Amsterdam UMC - location VU University Medical Center, Amsterdam, The Netherlands

Thank you for the opportunity to review this interesting paper. In this work, the age-specific incidence of the need for long-term care in Germany at the population level was estimated, based on national statistical data and following the illness-death model. The paper is well-written and seems technically sound. I have the following comments:

 1. In this paper, a method is described that avoids costly longitudinal data collections. However, the current approach also has drawbacks, as it mainly leads to descriptive data. For example, it is not possible to explain the incidence: no micro (individual characteristics) or macro (policy or environmental characteristics) factors can be explored in relation to the incidence. This is a limitation of the current study that could be acknowledged, in addition to the already mentioned data-related limitations (Page 6).  

 2. The approach chosen by the authors is one of several options to estimate the need for long-term care, such as simulation of scenarios. The method chosen is sound. However, I was wondering whether the authors have considered another option: linking representative cohort data to national statistical data? In many countries, this is possible, and it would enable even further exploration of factors driving the increase in need for long-term care. Could the authors reflect on whether this is an option in Germany? Or does GDPR prevents this?

 3. In the Introduction (Page 3), the authors write: “One way of estimating the incidence is to use the illness-death model”.  This raises the question: what are the other methods? Perhaps the authors could briefly mention these in their Discussion?

 4. Do I understand it correctly that for the assumption on the mortality rate ratio (R) a literature search was done that was not necessarily restricted to studies in Germany? (reference 22) The authors have carefully approached this by looking at a broad range with two scenarios, but how representative are data from other countries for the German situation?

 5. The focus of the paper is Germany. I miss some international context. For example, in the Discussion the results could be compared with similar data from other Western countries, if available. This would be important for policy makers, to see whether patterns are similar to other countries.

 6. Adding a definition of long-term care would be helpful for the Introduction. This probably covers residential care, but without further specification it could also cover informal care. Please clarify.

Is the work clearly and accurately presented and does it cite the current literature?

Yes

If applicable, is the statistical analysis and its interpretation appropriate?

Yes

Are all the source data underlying the results available to ensure full reproducibility?

Yes

Is the study design appropriate and is the work technically sound?

Yes

Are the conclusions drawn adequately supported by the results?

Yes

Are sufficient details of methods and analysis provided to allow replication by others?

Yes

Reviewer Expertise:

Geriatrics; Epidemiology

I confirm that I have read this submission and believe that I have an appropriate level of expertise to confirm that it is of an acceptable scientific standard.

# Peer review: Reviewer response for version 1

**DOI:** 10.5256/f1000research.142119.r166417

**Published:** 2023-03-17

**Authors:** Andreas Wienke

**Affiliations:** 1 Institute of Medical Epidemiology, Biometry, and Informatics, Martin-Luther-Universitat Halle-Wittenberg, Halle (Saale), Saxony-Anhalt, Germany

I would like to congratulate the authors for this excellent manuscript, which demonstrates how incidence of need for long-term care can be calculated based on prevalence, all-cause mortality and mortality rate ratio. A direct incidence estimation is impossible in Germany because of the lack of longitudinal data. Publicly available data are obtained from the Federal Statistics Office (all-cause mortality) and the nursing care statistics (prevalence). In the first part of the paper the relevant formula from previous work by the authors is given with respect to the relationship between incidence, prevalence, mortality, mortality rate ratio and remission. Based on this relationship the annual incidence is calculated.

 The paper is written in a very clear and sound style, I have only very minor remarks:

1. In the description of the data, a short explanation is missing regarding private and statuary care insurances in Germany. Do the data about prevalence cover only the situation of the statuary care insurance or also private insurances?
2. In Figure 1 it is difficult to distinguish the lines based on the legends.
3. I am not convinced by the decision to present the results of the calculations for the year 2015. Why not for the most recent year with data available?
4. I would prefer to use the wording of 'calculation' instead of 'estimation' (e.g. first line of Discussion).
5. As an outlook I recommend a more detailed discussion about a possible extension of the model including care levels. This would require the extension of the illness-death model with information about transitions between the care levels (is such data available?) and mortality rate ratios dependent on care level.

Is the work clearly and accurately presented and does it cite the current literature?

Yes

If applicable, is the statistical analysis and its interpretation appropriate?

Yes

Are all the source data underlying the results available to ensure full reproducibility?

Yes

Is the study design appropriate and is the work technically sound?

Yes

Are the conclusions drawn adequately supported by the results?

Yes

Are sufficient details of methods and analysis provided to allow replication by others?

Yes

Reviewer Expertise:

Epidemiology and Biostatistics

I confirm that I have read this submission and believe that I have an appropriate level of expertise to confirm that it is of an acceptable scientific standard.
